# Social isolation continued: Covid-19 shines a light on what self-advocates know too well

**DOI:** 10.1177/1473325020981755

**Published:** 2021-03

**Authors:** Ann Fudge Schormans, Sue Hutton, Marissa Blake, Kory Earle, Kevin John Head

**Affiliations:** School of Social Work, McMaster University, Hamilton, Canada; ARCH Disability Law Centre, Toronto, Canada; Ottawa, Canada; Toronto, Canada

**Keywords:** Social work practice, inclusion, social exclusion, self-advocacy, intellectual disability, ableism

## Abstract

Covid-19 has been an unprecedented time for social work as a profession and even more so for marginalized communities. This paper shares the reflections of three self-advocates (persons labelled/with intellectual disabilities engaged in advocacy and activism), a social worker, and a social work educator and researcher. It is intended as a rallying cry for social work to rethink how we deliver services to ensure that people who have already been made vulnerable through oppressive ableist practices and assumptions are not put at greater disadvantage at times like Covid-19. Hearing directly from self-advocates, we learn of their exclusion from pandemic planning, and of the ways that physical and social distancing protocols have worked to exacerbate the isolation, marginalization and inequities that people labelled/with intellectual disabilities have experienced over the years. We are called upon to more actively focus on advocacy efforts with people labelled/with intellectual disabilities to increase their involvement in planning, as well as access to supports, and to ensure that they do not remain “the left behind of the left behind” .

As has become undeniably clear, the Covid19 pandemic has not impacted people equally: long-standing social and structural inequities and injustices have significantly increased risk, harm, and disadvantage for some groups over others. The United Nations (2020) has reported that all people with disabilities have been hit particularly hard by Covid-19. Pandemic responses – marked by ableism – reveal that governments have failed to consider disabled persons’ living situations, support requirements, and daily realities in pandemic planning.

None of this has come as a surprise to us. It was anticipated. Each of us, in shared and separate ways, have for years (even decades) been speaking to the lack of acknowledgement and meaningful action towards addressing these inequities. What we also feared with the pandemic, was that people labelled/with intellectual disabilities would fare even worse. From our various vantage points, we see how the pandemic has brought to the surface, and intensified, the countless struggles that have persisted over the years for people labelled/with intellectual disabilities. We see too the alarming lack of attention being paid to this. People First of Canada (2020: 2), once again signaling the marginalized status of people living with the label ‘intellectual disability’ within even the larger disability community, argues that, during Covid-19, they are again “the left behind of the left behind.”

Thinking about people labelled/with intellectual disabilities as “the left behind of the left behind” risks overwhelming us as we are once more confronted with the extent and impacts of societal devaluation of people labelled/with intellectual disabilities. Faced again with the difficulty of achieving meaningful and lasting change. It also risks reinforcing their identification as only and always ‘victims’, thereby making invisible their actions during the pandemic. We have also heard the talk and wondered as to whether the pandemic might be a watershed moment, an awakening and a reckoning. The idea that by having disrupted so-called ‘normal’ ways of being and doing – more specifically, disrupting what many with privilege understand to be ‘normal’ – the pandemic offers an opportunity to work towards ‘real’ change, to address the inequities that have become so visible and, we hope, harder to ignore.

As a group, we are alarmed by what we are seeing, hearing and experiencing, particularly by the invisibility of people labelled/with intellectual disabilities in pandemic planning and responses. Kevin stresses the importance, the necessity, of educating others. We feel a practical urgency to share our experiences, our worries and our efforts, and to reflect on how to take advantage of this moment, with the recognition that for many communities, a return to ‘normal’ is neither needed nor desired. How might we (as self-advocates, SW practitioners, educators and researchers) and social workers more broadly, use our knowledge, experience, networks and skills going forward, to work towards change?

Hailing from Ontario, Canada, Marissa, Kory and Kevin identify as self-advocates labelled/with intellectual disabilities, each connected to local and national self-advocacy networks; Sue as a practicing social worker with an extensive history of advocacy and activism with self-advocates communities; and Ann as an educator and academic researcher with a lengthy social work practice background and extensive engagement with self-advocates in inclusive and activist research projects.

As a sobering start to our conversations, Marissa listed many interconnecting issues arising for her during the pandemic:

“First – is getting the support I need. That’s huge. Second is being able to socially distance. I use a wheelchair and can’t get off the sidewalk if someone doesn’t walk around and stay 6 feet away. Also, it’s really hard to communicate with staff who are wearing masks – I can’t understand what they’re saying or read their lips. It’s heartbreaking. Next is my managing my anxiety – my mental health. I just don’t know when this is going to end, what the future looks like. Staying healthy is another big worry – the disability community is at risk for Covid-19, and I’m nervous.”

The threat of contracting the virus and the impacts of safety protocols have taken a toll on Marissa, and she is worried, anxious and afraid. In similar and different ways, these feelings resonated with all of us.

We have heard from many self-advocates worried about contracting Covid-19, in the general context and, more particularly, from group home staff who are sometimes inconsistent with following safety guidelines, especially if travelling between different homes. We learned early into the pandemic that, due in large part to social devaluation and privatization of social services, people labelled/with intellectual disability – especially those in congregate care settings – have been put at greater risk of contracting and dying from Covid-19 (Turk et al., 2020). And that this did not have to happen. This is particularly frustrating given that conditions in these settings have been known for a long time. For Kory, having “advocated at the government level for changes over and over”, “being ignored is a slap in the face.” We all know, and care about, people living in these situations - the risks are real, people are getting sick, media reports are scary, and people we are hearing from are not feeling safe.

Ann and Sue have worked and shared relationships with people labelled/with intellectual disabilities for long enough to remember when services were less privatized, when more money for supports and staffing was available, when the aims of the de-institutionalization movement (while not sufficiently embraced) had more of an influence on policy and practice. The historical and contemporary contexts of systemic ableism are at play in the current range and complexity of pandemic impacts on people labelled/with intellectual disabilities. The question of “what to do” is complicated by the knowledge that what Marissa shared is just a sample of what people labelled/with intellectual disabilities are facing. Complicated by people’s variable support needs, existing/missing resources, and living arrangements. Ann and Sue, indeed, all social workers and people within and engaged with disability communities, are also living out the pandemic in different settings and circumstances. And systems are notoriously difficult to change. How then to intervene? This led us to the question of managing the tension between ‘safety’ and ‘well-being’, in particular, to addressing the impacts of lock-down and consequent Visitation Bans on people labelled/with intellectual disabilities.

No longer able to go to work, or attend his regular programs, Kevin has found staying at home and the social isolation following from that extremely difficult: “Oh it's so hard, it's hard you know? I miss [friends, family members, colleagues], I miss seeing people.” Attempting to cope with Covid-19 and control potential outbreaks, developmental services agencies were quick to follow Public Health protocols and implement strict Visitation Bans for residential programs. Other than a staff person who stops by his apartment regularly, Kevin sees no-one, and wonders why staff can’t visit more often, or stay longer. These bans are motivated by a desire that is difficult to fault – to keep people safe – a motivation that Kevin and other self-advocates we have heard from agree with. Nonetheless, these bans are disruptive and exacerbate the social isolation already common (if not, indeed, ‘normal’) in the lives of many people labelled/with intellectual disabilities. As people focused on removing systemic barriers to inclusion, the imposition of these protections on a community already experiencing unacceptable levels of exclusion has felt like a ‘sucker punch’.

What troubles us is that the manner in which bans have been imposed fails to consider disabled people’s needs, concerns and realities. As Kevin shared, significant impacts may follow from the disruption of routines, and the activities, support and social connection they bring. Combined with a not uncommon lack of access to technology, he struggles to occupy his time and is increasingly more anxious. Regular staff check-ins, while useful, cannot make up for what has been lost.

This is, however, not only about social isolation and loneliness. Visitation bans which restrict access to non-agency support persons have complicated Marissa’s life in many ways, necessitating extra work and much stress. Even food access has become an ordeal. Revealed is an apparent lack of understanding or dismissal of the importance of personal supports, and networks of support that are independent of residential agencies, to people labelled/with intellectual disabilities. And of the kinds and amount of support they can provide.

In another example speaking to this tension between risk, safety and well-being, Kory spoke about Public Health protocols and prohibitions against family members, carers or support persons accompanying disabled people in the hospital.

“My brother has intellectual disability and schizophrenia and asked me to support him at a hospital appointment during Covid-19. We contacted the hospital the day before to plan and prepare them but when we got to the hospital it was different. The screener asked my brother if he *really* needed support, pressuring him, saying ‘only one person was allowed in’. My brother was scared and gave in and said, “I guess not”. The advocacy didn’t kick in because we were nervous we’d be told we were causing a scene and be denied treatment. My brother felt so bad, that he did something wrong by bringing a support person to the hospital and went into the hospital without the support he needed.”

The potential consequences to people labelled/with intellectual disabilities of such restrictions are of great concern. Arguably, nobody fares well in these situations – not Kory’s brother, not Kory (worried and upset with himself for not advocating for his brother) and, likely, not the medical staff. The individual may be frightened, anxious in a hospital setting alone. Consequently, they may struggle to comply with treatment requests made by hospital staff unfamiliar with their support needs or communication style. This lack of ‘compliance’ may result in unnecessary medication or restraints. This type of experience, like the loss of social and supportive relationships for extended periods of time, may negatively impact physical and/or mental health.

Marissa and Kevin are looking for answers, wanting to know when and how this will end. Like many others denied access to communication technology, lockdown has meant Kevin spends too much time watching TV, following the news but not always understanding what he is hearing. What matters here is that televised news reports are rarely accessible for people labelled/with intellectual disabilities – this can increase anxiety. Kory cautions people to understand that some people labelled/with intellectual disabilities are experiencing “this whole new aspect of mental health.” Uncertain as to who to turn to, who to trust, some are reluctant to ask for help, worrying that “they’ll be ignored, locked up, drugged without reason”, or “that they won’t be believed.” And we are well aware that such worries are not unfounded – historical and contemporary examples abound.

Marissa was adamant that “People need to have access to supports. We needed to talk to other people about this.” Connecting online, while actively promoted as a solution to reduce social isolation, actually demonstrates the invisibility of people labelled/with intellectual disabilities in pandemic responses. As a social worker at ARCH Disability Law Centre, Sue coordinates Respecting Rights (RR), a project led by self-advocates with support from ARCH lawyers. When Covid-19 hit, RR in-person groups came to an abrupt halt. Sue, Marissa and Kory rapidly worked to get people online for meetings. It soon became clear that many self-advocates were left out. Most were without access to devices, support persons to teach them how to get online, or funds to pay for internet fees. In response, Sue and RR self-advocates launched the Get Connected campaign, reaching out to social media to help support people’s online access. While these moves were successful for some, the shift to online excluded far too many others. That people labelled/with intellectual disabilities were prevented at this time from learning about their rights, and that a campaign to secure funding to provide people labelled/with intellectual disabilities access to digital technology was necessary is, to our minds, inexcusable.

The pandemic has proven similarly disruptive for all involved in an inclusive research project underway for Ann and a large group of co-researchers labelled/with intellectual disabilities, in which project work was being done collaboratively, in face-to-face workshops. With pandemic lockdowns, the lack of access to technology, support persons, and funding for internet fees initially prohibited most co-researchers from participating in project work. Ann realized however that the work of the project was not what mattered most. Shifting her attention to supporting co-researchers, she set up telephone connections between co-researchers, and also linked them with project research assistants (RAs) based on relationships developed during the project. These phone calls are more personal and are separate from calls from RAs and other project members necessary to keep the project moving. Co-researchers have appreciated the social contact, as well as the opportunity to remain actively involved in project work, and having things to do. Nonetheless, such efforts are insufficient, and merely scratch the surface of what co-researchers need. In terms of the research project, made visible is the lack of funding for access provided by research funding bodies and the denial of the realities of social and economic inequities that can impact participation in research.

Social work practice, research and education has paid insufficient attention to people labelled/with intellectual disability (Fuld, 2020; Laws et al., 2010). Combined with the dangerous invisibility of people labelled/with intellectual disabilities in pandemic response planning, the need to take advantage of this moment to work towards change seems obvious.

From our perspective, if people labelled/with intellectual disabilities were incorporated into planning decisions as a matter of course, plans would likely look much different, still achieve their aims and, importantly, reduce differential risk. Their exclusion from planning can arguably be explained by disabling attitudes and assumptions of (in)ability and value. Kevin wonders who will read this paper and what they will do to make things better – that is why he participates in research and writing; he wants to hold to account people with the power to effect change, including social workers. Disrupting and changing ableist structures and systems, acknowledging that social work is implicated in these systems, is essential and, admittedly, an ongoing project but there are ways to move forward.

To begin, social workers must accept that a return to ‘normal’ is to be resisted – ‘normal’ has not worked in the past, has proven harmful, and will not be any better in the future. Social workers, as a group, must engage more directly *with* people labelled/with intellectual disabilities in advocacy and activist efforts towards change. They must, for example, join with them to speak against disabling poverty and educational discrimination that routinely denies people labelled/with intellectual disabilities digital literacy education, access to technology and supports, and development of alternative means of communicating. Recognize and attend to realities of social isolation, to the creation of opportunities and the development of networks of supports. Advocate for research funding that provides for disability access. Working *with* self-advocates to create greater public awareness, effect change in policy and practice in developmental, health, education and mental health sectors, and to make demands of governments all fall within the purview of social work and align with social justice aims.

We acknowledge that social workers have also experienced constraints during Covid-19. Nonetheless, we urge them to learn from the ways that people labelled/with intellectual disabilities have been left behind, further isolated and *made vulnerable*, not only by the inattention and inaction of governments during Covid-19, but also that of social work.
